# Contemporary challenges, needs and opportunities for emerging behavioral nutrition and physical activity researchers: a mixed-methods study

**DOI:** 10.1186/s12966-025-01748-1

**Published:** 2025-07-06

**Authors:** Brittany J. Johnson, Stephanie E. Chappel, Sarah Shaw, Emma R. Lawlor, Stephen Barrett, Kylie Wilson, Christine W. St Laurent, Hilary Caldwell, Brianne A. Bruijns, Sarah Burkart, Taylor J. Willmott, Daehyoung Lee, Simone J. J. M. Verswijveren

**Affiliations:** 1 College of Nursing and Health Sciences, Flinders University, Caring Futures Institute, Bedford Park, GPO Box 2100, Adelaide, South Australia 5001 Australia; 2 Centre for Quality and Patient Safety Research, Institute for Health Transformation, School of Nursing and Midwifery, Deakin University, Geelong, Australia; 3 Appleton Institute, School of Health, Medical and Applied Sciences, Central Queensland University, Adelaide, Australia; 4 MRC Lifecourse Epidemiology Centre, Faculty of Medicine, University of Southampton, Southampton, UK; 5 MRC Epidemiology Unit, University of Cambridge, Cambridge, USA; 6 School of Cardiovascular and Metabolic Health, University of Glasgow, Glasgow, UK; 7 Bendigo Health, Bendigo, VIC Australia; 8 Holsworth Research Initiative, La Trobe Rural Health School, La Trobe University, Bendigo, Australia; 9 College of Health Solutions, Arizona State University, Phoenix, AZ USA; 10 Department of Kinesiology, University of Massachusetts Amherst, Amherst, USA; 11 Healthy Populations Institute, Dalhousie University, Halifax, Canada; 12 School of Occupational Therapy, Western University, London, Canada; 13 Department of Exercise Science, Arnold School of Public Health, University of South Carolina, Columbia, USA; 14 Adelaide Business School, The University of Adelaide, Adelaide, Australia; 15 Department of Health Behavior and Nutrition Sciences, University of Delaware, Newark, USA; 16 Institute for Physical Activity and Nutrition (IPAN), Deakin University, Geelong, VIC Australia

**Keywords:** Early career researchers, Students, Career development, Nutrition, Movement behaviors, Sleep

## Abstract

**Background:**

Emerging researchers commonly navigate challenging and insecure working environments. Yet the impact on emerging behavioral nutrition and physical activity researchers is unknown. Hence, we sought to identify the contemporary challenges, needs, and opportunities for emerging behavioral nutrition and physical activity researchers.

**Methods:**

We employed a convergent mixed methods design, using an online survey. Participants completed socio-demographic questions, and rated the impact of personal and professional challenges, development needs with descriptive elaborations, and existing and desired professional development opportunities. Data analysis included thematic analysis of open-ended responses and descriptive statistics and multiple linear regressions of quantitative data. Integration of quantitative and qualitative data was through narrative and weaving.

**Results:**

Emerging researchers (*n* = 111, 57% graduate students) from over 20 countries participated. Synthesised results related to all four domains of the Researcher Development Framework. Specifically, we identified 8 themes relating to conducting research (domain 1); physical and mental health, and networking (domain 2); grant funding, and employment opportunities (domain 3); and leadership, supportive work networks, and communication with non-academic audiences (domain 4). Financial comfort was a predictor of both professional and personal development needs.

**Conclusions:**

Our study highlights the multiple challenges emerging researchers face, with increasing demands of collective efforts to support sustainable career development. Our findings serve as a foundation for promoting an inclusive and equitable research environment for emerging researchers. Though individual-level solutions may help, greater impact is likely from systemic changes to increase job security, career progression pathways and availability of ECR-specific funding.

**Supplementary Information:**

The online version contains supplementary material available at 10.1186/s12966-025-01748-1.

## Background

Research students and early career researchers (ECRs) (referred to collectively in this manuscript as emerging researchers) constitute the largest portion of the academic workforce, often face job insecurity and workplace challenges, negatively impacting their job satisfaction and intentions to remain in academia long-term [1–3]. The definition of ECRs varies widely [4], for this project we considered the following roles as ECRs, e.g., postdoctoral researcher, research fellow, faculty, research assistant with a PhD. In the field of behavioral nutrition and physical activity, emerging researchers are essential for driving long-term research efforts and advancing our understanding of healthy behaviors. An increasing number of emerging researchers leaving academia would have significant implications on future research progress in this field.

Emerging researchers generally face stressful, and often precarious, research and work environments. Many rely on government scholarships, supplemented by grant funding and sessional teaching [5, 6], often employed on short-term contracts with limited opportunities for ongoing academic positions [6]. Despite precarious employment, emerging researchers face high expectations around publication output and grant attainment [6]. Previous qualitative research has noted challenges among emerging researchers such as day-to-day management and work-life balance struggles, funding and employment stress, and handling multiple roles [1, 2, 7, 8].

The COVID- 19 pandemic impacted many aspects of society, including the research sector [9, 10]. Early research on the pandemic effects has acknowledged detrimental impacts for emerging researchers overall [11–14]. However, these studies were not specific to the unique circumstances of behavioral nutrition and physical activity researchers. Therefore, we sought to explore the contemporary challenges, needs, and opportunities for emerging behavioral nutrition and physical activity researchers.

## Methods

### Study design

The study followed a convergent mixed methods design, with quantitative and qualitative data collected in parallel, using an online survey with both quantitative and qualitative items [15]. We used a merging approach for integration [15] through narrative and weaving. Ethics approval was obtained from the Flinders University Social and Behavioural Research Ethics Committee (Approval ID: 5334). Reporting of this study was guided by the STROBE statement and SRQR checklists (see Additional file 1) [16, 17].

### Participants and recruitment

Participants were eligible if they self-identified as a student who conducts research (e.g., undergraduate, masters, PhD) or an ECR (e.g., postdoctoral researcher, research fellow, faculty research assistant with a PhD), and if their research related to behavioral nutrition, physical activity, sedentary behavior or sleep. There was no limit on geographical location.

Online survey recruitment took place during August to December 2022. The study was promoted through the International Society of Behavioral Nutrition and Physical Activity’s communication channels (e.g., social media, e-newsletters, webinars), and by other scientific societies (e.g., Asia–Pacific Society for Physical Activity, UK Society for Behavioural Medicine, European Obesity Community), universities and institutions (e.g., Indiana University Kinesiology Department). Recruitment also involved email invitations to authors’ existing networks and snowball sampling. To remunerate participants for their time, an optional randomised draw was conducted, offering the opportunity to win a one-year membership to International Society of Behavioral Nutrition and Physical Activity. Participants provided informed voluntary consent at the start of the online survey.

### Survey development and pilot testing

The survey was designed to be completed in one sitting of 20—30 min, including 13 demographic questions, an open-ended question defining an ECR and four sets of multiple-choice and open-ended questions to allow for an integrated mixed-methods approach (see Additional file 2). Participants were asked to quantitatively rate their experience of professional (9 items) and personal challenges (9 items) encountered since the COVID- 19 pandemic on a scale from (1) being no impact on your research/research career, to (10) being significant impact on your research/research career. Participants reported their current personal (13 items) and professional development needs (8 items), with (1) being no need for your research/research career and 10 being significant need for your research/research career. The list of potential challenges and needs were developed by the authors experience and engagement with students and ECRs as part of a professional society committee. For each category participants were able to add other additional items to the rating questions.

If participants quantitatively rated a challenge or need as ≥ 7/10, they were asked to elaborate on their challenges and/or needs from a qualitative perspective, including whether these experiences were from before and/or after the COVID- 19 pandemic. The survey also included five open-ended items asking participants to outline professional development opportunities they currently have, or would like to have, access to.

The survey was piloted with emerging researchers (n = 16) with expertise in health research to determine the readability of items, relevance of the list of challenges and needs, and to improve the survey structure and clarity of instructions provided. Minor changes were incorporated relating to the wording of instructions.

### Data analysis

#### Quantitative analysis

The total population size was unable to be determined, therefore the inferential statistics guided the sample size target. G*Power [18] was used to calculate the necessary sample size a priori to detect a medium effect size (0.15) with 0.80 power and an alpha level of 0.05 using up to seven predictors in multivariable linear regression models predicting professional and personal challenges and needs. A required sample size of 103 was determined.

All completed survey attempts were used in analyses, with descriptive statistics used to summarise findings. Ten-point Likert scale ratings of items within each need/challenge category were averaged and used as continuous outcome variables in four independent multiple linear regressions to explore predictors of each outcome. Responses marked as “Other”, where participants could enter and rank challenges not already identified, were not included in analyses (14 unique responses across the four categories). Six variables were included in each model as predictors: age, gender, race/ethnicity, current continent, level of financial comfort, and career stage. All analyses were performed using R statistical software [19].

#### Qualitative analysis

Open-ended survey responses were collated into individual transcripts for each participant. Qualitative analyses followed a pragmatic general inductive approach [20], given the data quality and project capacity. First, inductive coding was conducted in NVivo software [21], by three female authors who are ECRs with extensive qualitative research experience in the fields of diet and physical activity research (SS, SEC, ERL) who each independently coded a third of the transcripts. Inductive coding was selected to capture common challenges experienced by emerging researchers across different areas of their personal and professional lives (i.e. analysed as overall responses, not segmented by survey items). Inductive coding was most appropriate as the depth and breadth of the qualitative data varied across participants and survey items, making in depth analysis for some individual survey topics impossible. After coding 10 transcripts each, these three researchers met to discuss their initial interpretations of the data. After this, they independently coded the remaining transcripts, meeting regularly to discuss interpretations of that data and update, refine and finalise the coding. After all transcripts were coded, the researchers met again to decide on theme names that were accurate representations of the data. The coding and themes were checked by a second independent researcher (BJJ, ECR with qualitative research experience) with data recoded and the theme names reworded where appropriate.

#### Integration of quantitative and qualitative results

To aid translation of findings to university sector training we used the Vitae Researcher Development Framework [22] to structure presentation of the qualitative themes, supported by quantitative data ratings, combining data from professional and personal challenges, needs and opportunities. The Research Development Framework was developed in the UK and commonly used in UK and Australian universities to guide training of researchers including personal, professional and career development, particularly during the PhD period [22, 23]. The framework consists of four domains (each with 3 sub-domains): A) Knowledge and intellectual abilities, B) Personal effectiveness, C) Research governance and organisation, and D) Engagement, influence and impact. The framework has been used in similar studies [4, 24, 25]. Data were mapped to the framework by the first author (BJJ) and refined through workshopping with members of the research team (SJJMV, SBa, SS, CWL, ERL, HC, SBu, TJW).

## Results

### Sample characteristics

In total, 231 participants commenced the survey, with a 48% completion rate, resulting in 111 completed survey responses included in analysis. Participants primarily identified as women (78%) and White (72%) (Additional file 3). Approximately half of all participants were doctoral students (56%). Participants mainly represented North America, Europe, and Oceania. A variety of financial comfort levels were reported. All key research areas (i.e., physical activity, sedentary behavior, nutrition, sleep) were represented, with most participants (70%) declaring expertise in the physical activity area.

### Integrated results: professional and personal challenges, needs and opportunities

**Fig. 1:**
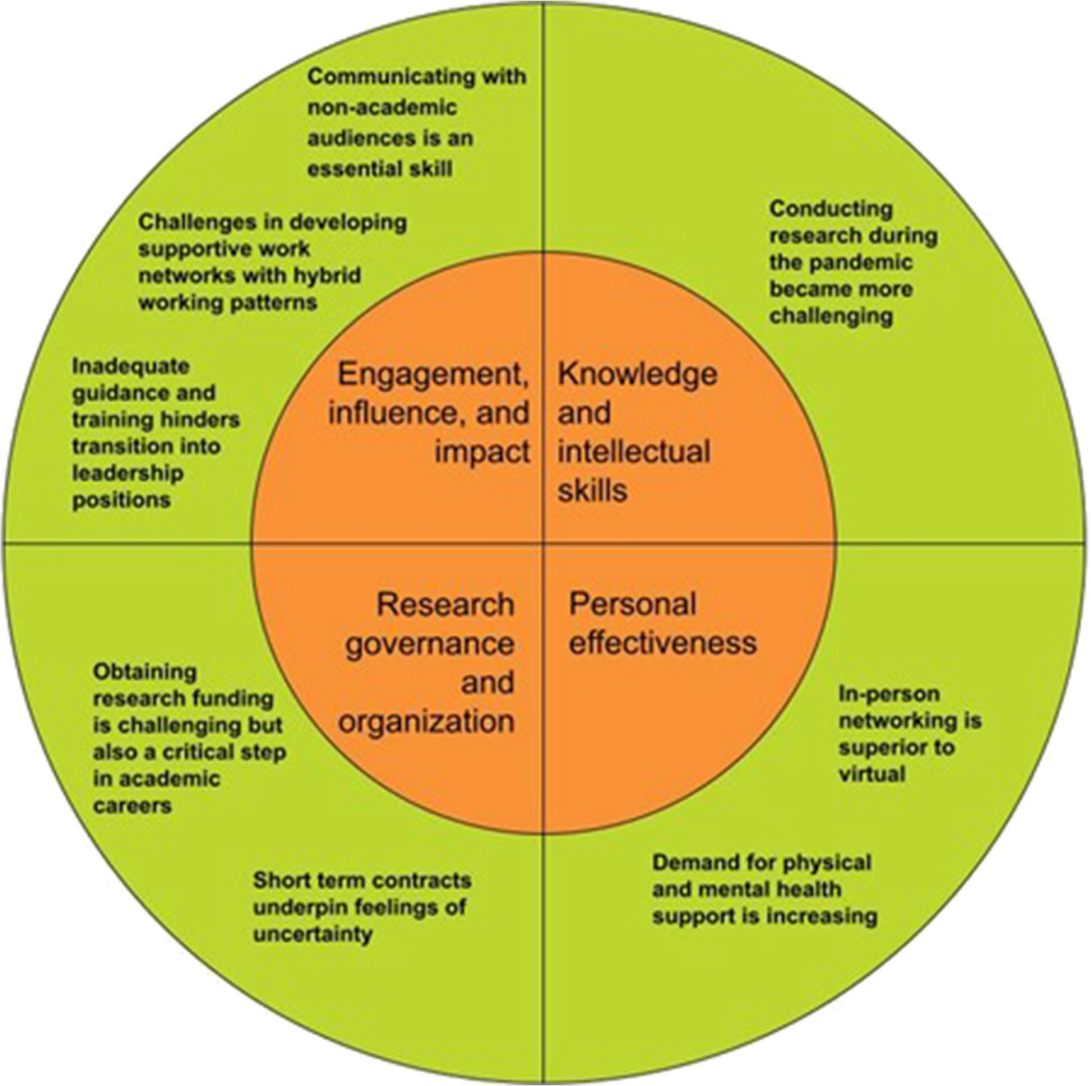
Qualitative themes identified by the research team based on the Vitae Researcher Development Framework [22]

This section presents the integrated findings from the quantitative ratings presented in Table 1 and the qualitative themes (Fig. 1, see Table 2 for exemplar quotes), relating to the professional and personal challenges, needs and opportunities for emerging behavioral nutrition and physical activity researchers.

**Table 1:** Measures of central tendency for professional and personal challenges, professional and personal development needs

**Items**	**Item score**				**Rated ≥ 7**	
	M	(SD)	Low	High	n	(%)
**Professional challenges (*n* = 110)**	**5.5**	**(1.8)**	**7**	**89**	**-**	**(-)**
Travel (*n* = 109)	7.7	(2.7)	1	10	82	(75.2)
Networking and collaboration (*n* = 110)	6.9	(2.3)	1	10	70	(63.6)
Conducting research (*n* = 109)	6.4	(2.8)	1	10	63	(57.8)
Remote working arrangements (*n* = 104)	5.2	(3.2)	1	10	42	(40.4)
Funding (*n* = 86)	5.2	(2.7)	1	10	26	(30.2)
Research culture (*n* = 105)	4.7	(3.0)	1	10	31	(29.5)
Time management (*n* = 105)	4.7	(3.0)	1	10	30	(28.6)
Employment (*n* = 92)	4.5	(3.3)	1	10	30	(32.6)
Supervision (*n* = 106)	4.4	(2.9)	1	10	28	(26.4)
Personal challenges (*n* = 109) (*Median, IQR*)*	***4.0***	(2.0)*	**2**	**80**	**-**	**(-)**
Caregiving (*n* = 60)	4.9	(3.8)	1	10	25	(41.7)
Mental illness (*n* = 78)	4.6	(2.9)	1	10	23	(29.5)
Being in quarantine/isolation (*n* = 95)	4.4	(2.9)	1	10	28	(29.5)
Loneliness/homesickness (*n* = 92)	4.3	(2.8)	1	10	25	(27.2)
Financial situation (*n* = 90)	3.4	(2.7)	1	10	14	(15.6)
Being infected with/exposed to COVID- 19 (*n* = 80)	3.3	(2.8)	1	10	14	(17.5)
Physical illness (*n* = 75)	2.8	(2.2)	1	10	7	(9.3)
Grief (*n* = 64)	2.7	(2.4)	1	10	6	(9.4)
Language (*n* = 53)	2.1	(2.3)	1	9	5	(9.4)
**Professional development needs (*n* = 110)**	**5.6**	**(1.9)**	**17**	**127**	**-**	**(-)**
Grant writing (*n* = 105)	7.0	(2.7)	1	10	61	(58.1)
Analysis	6.3	(2.5)	1	10	56	(50.9)
Networking and collaboration opportunities (*n* = 110)	6.3	(2.5)	1	10	54	(49.1)
Communicating research findings outside of academia (*n* = 109)	6.2	(2.6)	1	10	51	(46.8)
Scientific writing (*n* = 110)	6.0	(2.8)	1	10	49	(44.6)
Working with consumer researchers/end users (*n* = 96)	5.6	(2.9)	1	10	41	(42.7)
Research methods (*n* = 110)	5.5	(2.4)	1	10	40	(36.4)
Leadership (*n* = 102)	5.0	(2.8)	1	10	36	(35.3)
Project management (*n* = 102)	5.0	(2.9)	1	10	35	(34.3)
Communicating research findings within academia (*n* = 109)	5.0	(2.7)	1	10	32	(29.4)
Preparing job applications/resumes (*n* = 100)	4.9	(3.0)	1	10	35	(35.0)
Supervising research students (*n* = 91)	4.9	(2.6)	1	10	27	(29.7)
Managing research staff (*n* = 88)	4.5	(2.7)	1	10	25	(28.4)
**Personal development needs (*n* = 107)**	**4.5**	**(2.0)**	**4**	**74**	**-**	**(-)**
Career planning (*n* = 106)	5.9	(2.3)	1	10	43	(40.6)
Work-life balance (*n* = 107)	5.6	(2.7)	1	10	40	(37.4)
Stress and resilience (*n* = 104)	4.7	(2.8)	1	10	27	(26.0)
Financial planning (*n* = 101)	4.3	(2.8)	1	10	24	(23.8)
Physical and emotional health management (*n* = 103)	4.4	(2.8)	1	10	23	(22.3)
Sleep and routine management (*n* = 103)	4.2	(2.9)	1	10	22	(21.4)
Personal relationships with significant others (*n* = 98)	3.5	(2.7)	1	10	17	(17.4)
Language (*n* = 74)	3.1	(2.9)	1	10	10	(13.5)

All items on 10-point Likert scale, where 1 = no impact and 10 = significant impact on research/research career. Not every participant ranked all items, as there were no forced response and some items were not applicable. Bold values depict the average or median score across each of the four predetermined domains. * indicates data presented as median and IQR, rather than mean and SD.

**Table 2:** Exemplar participant quotes from qualitative data, by domain and theme

**Domain theme**	**Exemplar quotes**
***Domain A) Knowledge and intellectual abilities***	
Conducting research during the pandemic became more challenging	*“The 2020 COVID outbreak and March lockdowns meant that clinicians were moved to front-line roles and research was not a priority.”* (Participant 58, PhD student)
	“*Very difficult to recruit participants into studies, particularly having to rely on virtual means for recruitment, and more passive recruitment strategies.”* (Participant 76, ECR)
	*“We were still in the middle of participant recruitment and data collection and had to halt all research activities for several weeks, then drastically modify protocols to meet campus standards for conducting in-person work.”* (Participant 54, not reported)
	“*Data collection was postponed and needed to be done online now, which costed a lot of time that was initially planned to be devoted to writing and finishing my dissertation.”* (Participant 62, ECR)
***Domain B) Personal effectiveness***	
In-person networking is superior to virtual	“*Not being able to attend conferences or meet professors in the department casually affected my networking and collaboration as I was on the job market* … *Good collaborations for an ECR are extremely important and I believe they are fostered by meeting people in person.”* (Participant 36, ECR)
	*“I find so many of the virtual conference offerings to be almost futile. I can share my work on social media/online so readily and free, that investing all this money to present for* ~ *10–20 min seems pointless, especially since the engagement seems to be hampered online.”* (Participant 12, PhD student)
	“*It has become much easier to collaborate with my current network through video meetings (e.g., Zoom), but much more difficult to expand my network of collaborators.”* (Participant 104, ECR)
	“*For the past two years, I have noticed that different online platforms (e.g., Teams or Zoom) have given us researchers (or students) wide range of opportunities to collaborate. Although I am a big fan of face-to-face networking, I believe that we should take advantage of the technology in terms of global, cost-effective meetings.”* (Participant 108, PhD student)
	*“I would benefit from additional opportunities to do so [network], as well as opportunities to learn more about how to successfully network with other researchers.”* (Participant 71, Masters student)
Demand for physical and mental health support is increasing	“*The lockdowns (quarantine) had a significant toll on my mental health, which impacted my research.”* (Participant 32, ECR)
	“*…being in quarantine and isolation made me less productive in my research as there was a lack of accountability in staying productive at home.”* (Participant 34, PhD student)
	*“I haven't been able to go home for two years. The homesickness has been impacting my mental health.”* (Participant 103, PhD student)
	“*Isolation because of covid exacerbated previous depression and anxiety. This made it difficult to focus on my research including finishing my PhD and starting a postdoc.”* (Participant 87, ECR)
	“*I would like resources for managing the stress of a PhD.”* (Participant 38, PhD student)
	“*Would like more transparent discussion and workable strategies for combating stress and building resilience in the workplace – this was needed prior to the pandemic, but pandemic definitely increased feelings of stress.”* (Participant 76, ECR)
	“*…find it hard to maintain good work-life balance with the expectations I place on myself, that are placed on me by my superiors, and that are required to be competitive for external funding and job applications.”* (Participant 76, ECR)
***Domain C) Research governance and organisation***	
Obtaining research funding is challenging but also a critical step in academic careers	“*Obtaining grant funding is integral to career development as both Co-I and PI. This has been even harder during the pandemic with reduced collaboration, networking and research funding opportunities.”* (Participant 68, ECR)
	“*Funding is inextricably linked to employment and career progression; this in turn is challenging due to the precarity of research projects, fewer funding opportunities available particularly in a highly competitive climate. Moreover, duration, workload, and precarity of contracts pose further challenges in relation to funding opportunities for ECRs.”* (Participant 67, ECR)
	“*Difficulty in securing funding, spending a large proportion of my time writing grants that are not successful. This was pre and post-COVID, but is only getting worse since COVID with University funding being reduced.”* (Participant 57, ECR)
	*“I am currently writing my first grant applications. Workshops or support programs at our university are rather scarce.”* (Participant 107, PhD student)
	*“[You] need training and experience in grant writing to be able to bring the research career forward and to keep finances of current position.”* (Participant 69, PhD student)
Short term contracts underpin feelings of uncertainty	“*Getting very stressed about my career and where I will go now that I'm getting closer to the end of my PhD.”* (Participant 40, PhD student)
	*“Contracts have been short, casual, 6-month fixed term since the start of the pandemic. This has been really awful.”* (Participant 79, ECR)
	*“I have no idea where I will be able to be employed after I finish my PhD. I would like it to be some type of ‘safe’ full time job. I have not been able to find that up until now.”* (Participant 25, PhD student)
	“*Having a fixed-term contract was nerve-wracking because I had no idea of whether I would secure anything more when it ended, but luckily I got a 2nd 1-year contract which allowed me to continue… Being 2/3 of the way through the 2nd post-doc is again nerve-wracking…”* (Participant 101, ECR)
	*“I ended up being unemployed for 7 months following the end of my PhD because of COVID. I was not prepared to stay this long without pay and it took a big toll on my finances and mental health.”* (Participant 19, PhD student)
***Domain D) Engagement, influence and impact***	
Inadequate guidance and training hinders transition into leadership positions	*“At the beginning of the research career, you need to jump at some point to start managing a project. But it is not an easy task, and is not something you are taught in the University (although, it would be nice). This is one of the things I have been struggling with as an ECR, and I would like to learn more about.”* (Participant 88, ECR)
	*“When it comes to research-related project management—especially with large, slippery and geographically scattered groups of fellow researchers—I have much to learn. With regards to financial management of research projects, I don't know zero—though that was the case just a year and a half ago—but I would have to rate my knowledge as very close to zero. This need has arisen post-Covid as I stepped into the research world in a far bigger way than during my pre-Covid solitary PhD journey.”* (Participant 101, ECR)
Challenges in developing supportive work networks with hybrid working patterns	*“Connect with a successful faculty member who has experienced the same struggles you experience (racism, classism, sexism, *etc*.). Academic spaces are incredibly gate-kept — find an advocate who is flourishing despite these systemic challenges. There is power in community.”* (Participant 95, ECR)
	*“I have monthly meetings with my supervisors about my progress. My supervisors are also very accessible to ask questions or seek feedback in between. In our research group, I was also assigned a mentor (a postdoctoral researcher). Finally, I work in a group with very nice colleagues who are also always willing to help where needed.”* (Participant 60, PhD student)
	*“I couldn't just pop into a mentor's office and ask a question, but rather to email or text them, which felt more artificial and less personal especially if the question was about a significant research issue.”* (Participant 42, PhD student)
	“*The most challenging aspect, however, was not being able to see and catch up with colleagues in-person. Monday morning meetings, SUAW [shut up and write] sessions, and Friday happy hour were moved online during COVID. Over time attendance at these events dropped to virtually no one attending.”* (Participant 84, ECR)
	*“I experienced negative mental health issues due to bullying in my supervisory team and was unable to access support from the university. This was further exacerbated by two years of isolation as the university remained closed for the final leg of my PhD.”* (Participant 39, ECR)
	*“…mental health not being of significance to my faculty and/or mentor made my mental health unfortunately deteriorate.”* (Participant 42, PhD student)
Communicating with non-academic audiences is an essential skill	*“We as researchers need to think about how our research questions and interventions are relevant to the public and the target audience, and working with them more can only help that.”* (Participant 31, ECR)
	*“I had an emphasis before covid in summarizing research findings for participants and it really seems like one way to rebuild public trust after covid is to improve how we communicate what we do, why it matters, and what our findings mean in a way people can understand.”* (Participant 87, ECR)
	*“I find it hard to translate complex findings to outside academia. My results are often dry.”* (Participant 100, ECR)
	*“I would like to learn how to better communicate findings with the public & get them excited by our findings.”* (Participant 32, ECR)

#### Knowledge and intellectual abilities

Conducting research during the pandemic became more challenging was identified as a theme related to *Domain A) Knowledge and intellectual abilities*.

##### Conducting research during the pandemic became more challenging

In the quantitative findings, conducting research was the third highest rated professional challenge (mean 6.4, SD 2.8), with over half of respondents rating it a challenge (58%, rated ≥ 7 of 10). Similarly, data analysis (a part of conducting research) was the second highest professional development need (mean 6.3, SD 2.5, 51%, rated ≥ 7 of 10). In long-form written responses, emerging researchers shared further context about the difficulties they encountered in recruiting participants and conducting research, attributing these difficulties to pandemic-related restrictions. Recruitment may have worsened since COVID- 19 with pandemic-related restrictions still being enforced including school closures, hospital priorities, and remote working. The pandemic also required a shift to online recruitment and data collection, limiting the field of potential participants. Respondents reported how the pandemic also impacted research plans and delayed progress in degrees and studies.

#### Personal effectiveness

Two themes were generated that related to *Domain B) Personal effectiveness*: In-person networking is superior to virtual; and Demand for physical and mental health support is increasing.

##### In-person networking is superior to virtual

Networking as a professional challenge and development need was combined with collaboration as an item, and rated as a challenge (≥ 7 out of 10) by 64% of participants (mean 6.9, SD 2.3), with 49% of participants rating the need for more networking and collaboration opportunities (mean 6.3, SD 2.5). Related to networking, travel was the highest rated professional challenge (mean 7.7, SD 2.7; ≥ 7 out of 10 by 75%). Participants described collaboration and networking as essential for a future career; “*Most jobs seemed to be obtained through connections, which makes networking and collaboration really critical.* (Participant 18)”. Noting many barriers to networking due to COVID- 19, including not being able to attend conferences, limited face-to-face networking opportunities and difficulties with online networking. These barriers made it difficult to make new connections and potential collaborations.

Despite the challenges resulting from the pandemic, some described the positives of increased virtual opportunities. Participants described using online platforms to make connections and work closely to collaborators remotely, although for some this still had drawbacks.

Participants also described needing more opportunities to network face to face and stated they would value opportunities for formal training to learn how to network effectively.

##### Demand for physical and mental health support is increasing

The most highly rated personal challenges and development needs align with *Domain B Personal* *effectiveness*. The highest ranked personal challenges were caregiving (median 4.9, IQR 3.8; 42% rated ≥ 7 of 10), mental illness (median 4.6, IQR 2.9; 30% rated ≥ 7 of 10), and being in quarantine/isolation (median 4.4, IQR 2.9; 30% rated ≥ 7 of 10). The highest ranked personal needs were career planning (mean 5.9, SD 2.3; 41% rated ≥ 7 of 10), work-life balance (mean 5.6, SD 2.7; 37% rated ≥ 7 of 10), and stress and resilience (mean 4.7, SD 2.8; 26% rated ≥ 7 of 10). Participants described a bi-directional relationship between their physical and/or mental health and their work. These bidirectional impacts were heightened during the pandemic.

Some participants reported disruptions in social connections from pandemic restrictions impacted their mental health.

Some physical and/or mental health challenges directly related to COVID- 19 restrictions. For others, being physically inactive and isolated during quarantine negatively impacted the mental health and productivity of individuals. Participants recognized the need to maintain good health to enhance productivity and work efficiency, and prioritising physical and mental health. Participants also described needing strategies to handle stress and build resilience in the workplace.

Emerging researchers often reported compromising their work-life balance to achieve the high expectations, and the remote work arrangements during the pandemic blurred the work-life boundary. PhD students felt there was an expectation to work long hours, making it hard to fulfil caregiving responsibilities. ECRs also described a pressure to constantly develop new skills, including statistical analysis, and it is challenging to keep up with the pressure to learn more.

#### Research governance and organisation

Two themes aligned with *Domain C) Research governance and organisation*; Obtaining research funding is challenging but also a critical step in academic careers: and Short term contracts underpin feelings of uncertainty.

##### Obtaining research funding is challenging but also a critical step in academic careers

Despite the challenges of obtaining funding being discussed, only 30% of participants rated funding as a challenge (≥ 7/10; mean 5.2, SD 2.7). Grant writing on the other hand, was rated as a professional development need by 58% of participants (mean 7.0, SD 2.7). Securing research funding was a challenge discussed by many participants, highlighting changes brought about by the pandemic negatively influenced their capacity to secure the funding necessary to help establish their academic career. Such challenges existed pre-pandemic, however issues such as time constraints during the pandemic reportedly heightened some of these issues.

Challenges securing research funding closely linked to the challenges associated with fixed and short-term contracts.

Participants noted being unsuccessful in previous grant applications and were cognisant of the time required in submitting applications. Participants also highlighted challenges in securing small, ECR-focused funding that were seen as a first step to obtaining larger, more competitive bids. Many participants felt they lacked the necessary skills to write a competitive funding application. Adequate training in these important skills, and support to write funding applications was difficult to find and many respondents felt this has been made more challenging through the pandemic.

##### Short term contracts underpin feelings of uncertainty

Employment opportunities and job security were major concerns described by participants. Participants felt that the uncertainty associated with short term contracts was likely to persist for some time if they remained in this line of work. The pandemic highlighted how insecure working arrangements were for ECRs, and some respondents reported losing their jobs during this time. Fixed and short-term employment and uncertainty over long-term career pathways were described as sources of stress.

#### Engagement, influence and impact

Three themes generated related to *Domain D) Engagement, influence and impact*: Inadequate guidance and training hinders transition into leadership positions; Challenges in developing supportive work networks with hybrid working patterns; and Communicating with non-academic audiences is an essential skill.

##### Inadequate guidance and training hinders transition into leadership positions

As a professional development need, leadership was rated mean 5.0 (SD 2.8), with 36% of participants rating this as ≥ 7 (out of 10). Becoming a leader in the field was reported as crucial for a successful research career but also a challenge for emerging researchers.

Participants highlighted they wanted to improve their leadership skills, including capacity building, policy change, managing projects, and gaining research funds. They perceived supervising research students was essential for academic success and expressed the desire to gain skills and experience as (co-)supervisors, and the ability to lead and deliver projects on time and within budget as crucial for securing employment and funding. Many respondents expressed the need for further support, training and development in project management, especially for larger research projects and coordinating longitudinal studies, and for delegating tasks and balancing responsibilities. These support needs provide opportunities to enhance leadership from early in the research career through investment in project management training and career planning at the beginning of a PhD degree.

##### Challenges in developing supportive work networks with hybrid working patterns

Participants expressed the importance of connecting and collaborating with experienced faculty members and a supportive research community. During the pandemic, the importance of having a supportive network was really highlighted to participants as they needed help to navigate transitions in projects or PhDs due to the impact of COVID- 19. Receiving informal mentorship from colleagues was valued or was desired.

Participants highlighted a shift to remote work during the pandemic resulted in decreased frequency of meetings, reduced informal communication with supervisors/line managers or feedback. Many participants reported the impact of lack of in-person interactions with supervisors and colleagues that are important for effective communication, relationship development and rapport. Negative experiences of supervision were described with some participants experiencing a lack of support and/or bullying from line managers, or feeling the strains of academic life not only directly but also indirectly through their supervisor.

##### Communicating with non-academic audiences is an essential skill

Participants rated professional development needs of communicating research findings outside of academia as a mean of 6.2 (SD 2.6, 47% rating ≥ 7 out of 10), and working with consumer researchers/end users as a mean 5.6 (SD 2.9, 43% rating ≥ 7 out of 10). Several participants noted the heightened importance of communication with lay audiences to combat misinformation and distrust in science that increased due to COVID- 19. There were several challenges raised related to science communication, predominantly difficulties in communicating with non-academic audiences and a lack of training and support in such communication skills.

### Socio-demographic predictors of challenges and needs

Additional file 4 present the models examining professional development needs (*F*(86) = 1.99, *p* < 0.05, Adj. *R*^*2*^ = 0.13), and personal development needs (*F*(84) = 1.72, *p* < 0.05, Adj. *R*^2^ = 0.10). Participant financial situation was a significant independent predictor of professional development needs, indicating that participants who reported being more financially comfortable had fewer unmet professional development needs (β = − 0.33, *p* < 0.05, 95%CI − 0.65,− 0.01). A similar relationship was identified in the personal development needs model, where participants who reported being more financially comfortable had fewer unmet personal development needs (β = − 0.66, *p* < 0.01, 95%CI − 1.03,− 0.30). The linear regression models examining predictors of professional challenges and personal challenges did not meet model fit requirements (Additional file 4).

## Discussion

We identified eight themes that incorporated different elements of challenges, needs and opportunities relating to conducting research, physical and mental health, networking, grant funding, employment opportunities, leadership, supportive work networks, and communication with non-academic audiences. The pandemic exacerbated the challenges and needs for emerging researchers. Financial comfort was a predictor of both professional and personal development needs. Although writing groups, mentoring, and networking were identified by emerging researchers as pathways to facilitate career development in the short-term, systemic change is needed to reduce several of the challenges faced by emerging researchers, such as high stress and poor mental health, job insecurity and low grant funding success to ensure greater retention among future generations of emerging researchers.

For our participants, the pandemic illuminated many long-standing deficiencies within and across universities such as financial stress, job insecurity, and a “cut-throat” academic culture fuelling poor work-life balance (including inequities for carers) and mental health. Work is underway by organisations such as The Royal Society and Wellcome Trust to review and improve the research culture more broadly [26, 27]. Aligned with our findings, navigating multiple roles and maintaining work-life balance is another long-standing challenge for emerging researchers [2, 3, 7, 8]. The challenges of an uncertain job market, work/role overload and financial concerns have a cumulative effect on the mental health of emerging researchers [5]. With many universities and research institutions re-designing their policies after the pandemic, now is an ideal time to address many of the action items and build a more sustainable model for academia.

We found lower financial comfort was associated with both higher professional and personal development needs and was the only significant socio-demographic predictor in our sample. It is well documented that financial uncertainty triggered by the pandemic and related institutional revenue loss endangered the career prospects of ECRs [28, 29]. Most emerging researchers are employed on casual or fixed-term contracts, putting them in the most vulnerable position as the increasing number of universities experienced layoffs and hiring freezes [30–32]. Uncertainty regarding employment contracts and associated career trajectories was highlighted by our participants, and is a consistent concern amongst emerging researchers more broadly [3, 8]. For the greatest impact, changes to institutional systems such as improvements in job security and career progression pathways are likely to be more important, and further consultation with a wider range of stakeholders capable of assisting in the implementation of supportive interventions is recommended [33, 34].

Moreover, there is a lack of university-level or external targeted grant opportunities available for ECRs, and those that exist are often disproportionately geared toward ECRs at the end of eligibility bracket. Recent Medical Research Future Fund schemes for Australian ECRs had as low as a 2.5% success rate [35] and similar challenges can be found across the globe [36]. Research with other disciplines suggested that emerging researchers can greatly benefit from early exposure to grant writing as part of their PhD/independent research training [37, 38]. A study explored the impact of grant-writing support in American ECRs and reported successful program outcomes (e.g., increased success rate of applications), as a result of accountability structures and focused mentoring strategies [38]. Given the instrumental role of emerging researchers, academic institutions and research societies should provide grant writing training and support in early career stages, as well as advocate for and diversify career planning with PhD students and provide ECRs with more funding opportunities. Further, national health and scientific research agencies and research institutes should be more intentional about including and recognizing emerging researchers in grant teams as it often results in much-needed learning opportunities, compelling track records, and competitiveness in the job market. Funders could further support emerging researchers through strategic priorities and revising grant criteria to support embedding ECRs in all research teams in funding applications, to improve ECR development and productivity. Examples of similar strategic priorities are seen to address gender equity in grant schemes [39].

Strengths of this project included the research team is a diverse group of emerging researchers; this first-hand experience ensured survey topics were highly relevant and aided data interpretation. The exclusive focus on behavioral nutrition and physical activity meant results are applicable and actionable to those working in this field, rather than being too broad. Further, the mixed methods design enabled exploration into the quantitative results and participants at various career stages responded, providing a comprehensive representation. Substantial effort was made during recruitment to obtain a diverse sample, including through personal and professional networks to low- and middle-income countries (e.g. Global Diet and Activity Research Network). While majority of the sample identified as being white, a quarter identified as being Hispanic or Latino, Asian, Black or another race/ethnicity. Unsurprisingly, few participants were on a career break/disruption when completing the study, yet this does not reflect the number of participants who are a parent/career, with 55% of participants rating the personal challenge of caregiving. The survey was only available in English and may not have reached, hence reflect, all eligible emerging researchers. For the open-ended questions, there were limited responses and varying detail provided, restricting the richness of qualitative results. This was particularly apparent for the items relating to opportunities. The large sample size, limited response depth and project capacity meant a pragmatic approach to qualitative analysis was implemented.

## Conclusions

Emerging behavioral nutrition and physical activity researchers face many challenges professional and personal challenges. Short and fixed-term employment and absence of career progression pathways were, and continue to be, sources of stress and mental health concerns. Individual-level approaches may help address some of these challenges, though greater impact is likely from systemic changes to increase job security, career progression pathways including availability of ECR-specific funding. Our findings serve as a call to action for universities, funders, and societies to enhance support for emerging researchers as they navigate this unique career stage in the current climate. Actively engaging and supporting emerging researchers as they transition to the workforce is essential to building a sustainable model for the research sector and its utility in both academic and non-academic settings.

## Supplementary Information

Supplementary Material 1.

Supplementary Material 2.

Supplementary Material 3.

Supplementary Material 4.

## Data Availability

A de-identified data set will be made available for other research projects through the Repository of Open Access Data Sets (https://open.flinders.edu.au/).

## Abbreviations

ECR: Early Career Researcher
ISBNPA: International Society of Behavioral Nutrition and Physical Activity
NESI: Network of Early Career Researchers and Students of ISBNPA
