# Supplementary material for: Contemporary challenges, needs and opportunities for emerging behavioral nutrition and physical activity researchers: a mixed-methods study
Source: Int J Behav Nutr Phys Act. 2025 Jul 6;22:94. doi: 10.1186/s12966-025-01748-1 (PMC12232601; doi:10.1186/s12966-025-01748-1)
Supplement: Supplementary file 2 — Supplementary Material 2. [file 12966_2025_1748_MOESM2_ESM.docx]

**Additional file 2: Copy of the survey**

**Start of Block: Captcha Verification and Eligibility**

Welcome 

Thank you for your interest in the **"Challenges, needs and opportunities for emerging behavioral nutrition and physical activity researchers: in (post-)pandemic times"** study. 

Please complete a few questions to confirm you are eligible to participate. If eligible, you will be presented with more information about the project and have the opportunity to consent to participate in the study.

check Before entering the survey, please confirm you are not a robot:

| Page Break |  |
| --- | --- |

| 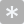 |
| --- |

screening_1 Are you a student who conducts research (e.g. undergraduate, masters, PhD) or an early career researcher (e.g. postdoctoral researcher, research fellow, faculty, research assistant with a PhD)?

- Yes (1)
- No (2)

| Page Break |  |
| --- | --- |

| 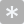 |
| --- |

screening_2 Does your research relate to physical activity, sedentary behavior, sleep, and/or behavioral nutrition?

- Yes (1)
- No (2)

**End of Block: Captcha Verification and Eligibility**

**Start of Block: Letter of Information and Consent**

Information Sheet Welcome and thank you for taking part in this research project.

This project is led by the Network of Early Career Researchers and Students of ISBNPA (NESI), the early career researchers (ECRs) and students community within the International Society of Behavioral Nutrition and Physical Activity (ISBNPA).

The aim of the project is to understand the challenges, needs, and opportunities for emerging behavioral nutrition and physical activity (including sedentary behavior and sleep) researchers in (post-)pandemic times.

We seek student (e.g. undergraduate, masters, PhD) and ECR (e.g. post-doctoral researchers, research fellows) perspectives of the current and potential research climate in a (post-)pandemic world. This survey will ask you questions about yourself, your research, and the challenges, needs, and opportunities you are experiencing. We are planning to publish this work as it could help advocacy and benefit researchers and research institutes in this field.

This survey includes **four sections:**
**Part 1 and 2** will ask you about your personal and professional challenges and needs
**Part 3** will ask you to share research opportunities that you perceive now and in the near future
**Part 4** will ask about your demographics

We have provided instructions in each section to help you complete the survey. You may advance or go back to earlier responses using the buttons provided at the bottom of the screen.

Before beginning the survey, it is important that you have a full understanding of what is involved in participation.

 **Please click the** [link here](https://qualtrics.flinders.edu.au/CP/File.php?F=F_39JM7yW224FcXJQ) **to open the Participant Information Sheet, which provides details and background information about this research.**

It is important to read the Participant Information Sheet, as it clearly describes the research, why we are doing it, what you can do if you would like to withdraw, how your information is protected and how you can contact us if you need further information. If you would like to, you can save a copy for your records.
On completion of the survey, you will have the chance to enter the prize draw to win a 1 year International Society of Behavioral Nutrition and Physical Activity membership.

**Participation in this survey is voluntary and information provided is confidential**. The survey will take around **20-30** minutes to complete, and is best completed on a laptop / desktop device. If you have any concerns about this study, please contact the research team via email: [nesi.isbnpa@gmail.com](mailto:nesi.isbnpa@gmail.com?subject=NESI%20Project).

**Consent Statement**
I have read and understood the information about the research, and I understand I am being asked to provide informed consent to participate in this research study.

I understand that I can contact the research team if I have further questions about this research study.

I am not aware of any condition that would prevent my participation, and I agree to participate in this project.

I understand that I am free to withdraw at any time during the study.

I understand that I can contact Flinders University’s Research Ethics & Compliance Office if I have any complaints or reservations about the ethical conduct of this study.

I understand that my involvement is confidential, and that the information collected may be published. I understand that I will not be identified in any research products.

**Please select one of the options below.**

- I agree to the above statements and give my full consent to participate in this study (1)
- I do not agree to the above statements and do not wish to take part in this study (2)

*Skip To: End of Survey If Consent Statement I have read and understood the information about the research, and I understand... = I do not agree to the above statements and do not wish to take part in this study*

*Display This Question:*

*If Consent Statement I have read and understood the information about the research, and I understand... = I agree to the above statements and give my full consent to participate in this study*

| 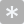 |
| --- |

Further Consent I further consent to (please select all that are relevant):

- Completing a questionnaire (1)
- Sharing my de-identified data with other researchers (2)
- My data and information being used in this project and other related projects for an extended period of time (no more than 5 years after publication of the data) (3)
- Being contacted about other research projects (4)

**End of Block: Letter of Information and Consent**

**Start of Block: ECR Definition**

information **Thank you for agreeing to participate in this study. Your insights are greatly appreciated.**There are numerous definitions of research career stages, with definitions not just differing between countries, but even differing between institutions or disciplines. The next question helps us to understand the range of definitions. There are no right or wrong answers.

Q1 How would you define an Early Career Researcher? Please provide as much detail as possible.

________________________________________________________________

________________________________________________________________

________________________________________________________________

________________________________________________________________

________________________________________________________________

**End of Block: ECR Definition**

**Start of Block: Challenges**

Information **Part 1. Challenges**

The next questions focus on challenges you have faced both professionally and personally. When referring to personal challenges, think of these as challenges that impact your research / professional capacity.

Q2 Please rate your experience of the following **professional** challenges you have encountered since the COVID-19 pandemic was declared in March 2020, with 1 being no impact on your research / research career, and 10 being significant impact on your research / research career.

|  | No impact | Significant impact | Not Applicable |
| --- | --- | --- | --- |

|  | 1 | 2 | 3 | 4 | 5 | 6 | 7 | 8 | 9 | 10 |
| --- | --- | --- | --- | --- | --- | --- | --- | --- | --- | --- |

| Conducting research (e.g., recruitment, data collection, intervention delivery, analysis, writing etc.) () | 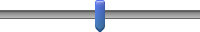 |
| --- | --- |
| Networking and collaborations (e.g., building, maintaining) () | 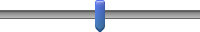 |
| Funding (e.g., availability and/or accessibility of funding opportunities, success rates etc) () | 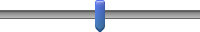 |
| Employment (e.g., casual alongside studying, post PhD etc.) () | 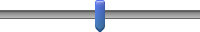 |
| Remote working arrangements (e.g., access to universities/institutes, isolation etc.) () | 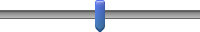 |
| Travel (e.g., regionally, nationally, or internationally for meetings, conferences etc) () | 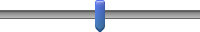 |
| Supervision (e.g., type, frequency, etc) () | 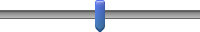 |
| Time management (e.g. time pressure to complete degree/projects on time or within funding window) () | 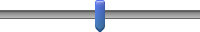 |
| Research culture (e.g., distrust related to science, changing landscape) () | 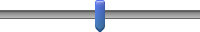 |
| Other: please state () | 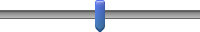 |

| Page Break |  |
| --- | --- |

*Display This Question:*

*If Please rate your experience of the following professional challenges you have encountered since t... [ Conducting research (e.g., recruitment, data collection, intervention delivery, analysis, writing etc.) ] >= 7*

Q3a **As you rated this item highly please provide additional details to help us understand this challenge**

Please describe your experience of **Conducting Research** challenges on your research / research career. Please include whether the challenge was experienced before or after the COVID-19 pandemic was declared in March 2020 (or both).

________________________________________________________________

________________________________________________________________

________________________________________________________________

________________________________________________________________

________________________________________________________________

| Page Break |  |
| --- | --- |

*Display This Question:*

*If Please rate your experience of the following professional challenges you have encountered since t... [ Networking and collaborations (e.g., building, maintaining) ] >= 7*

Q3b **As you rated this item highly please provide additional details to help us understand this challenge**

Please describe your experience of **Networking and Collaboration** challenges on your research / research career. Please include whether the challenge was experienced before or after the COVID-19 pandemic was declared in March 2020 (or both).

________________________________________________________________

________________________________________________________________

________________________________________________________________

________________________________________________________________

________________________________________________________________

| Page Break |  |
| --- | --- |

*Display This Question:*

*If Please rate your experience of the following professional challenges you have encountered since t... [ Funding (e.g., availability and/or accessibility of funding opportunities, success rates etc) ] >= 7*

Q3c **As you rated this item highly please provide additional details to help us understand this challenge**

Please describe your experience of **Funding** challenges on your research / research career. Please include whether the challenge was experienced before or after the COVID-19 pandemic was declared in March 2020 (or both).

________________________________________________________________

________________________________________________________________

________________________________________________________________

________________________________________________________________

________________________________________________________________

| Page Break |  |
| --- | --- |

*Display This Question:*

*If Please rate your experience of the following professional challenges you have encountered since t... [ Employment (e.g., casual alongside studying, post PhD etc.) ] >= 7*

Q3d **As you rated this item highly please provide additional details to help us understand this challenge**

Please describe your experience of **Employment**challenges on your research / research career. Please include whether the challenge was experienced before or after the COVID-19 pandemic was declared in March 2020 (or both).

________________________________________________________________

________________________________________________________________

________________________________________________________________

________________________________________________________________

________________________________________________________________

| Page Break |  |
| --- | --- |

*Display This Question:*

*If Please rate your experience of the following professional challenges you have encountered since t... [ Remote working arrangements (e.g., access to universities/institutes, isolation etc.) ] >= 7*

Q3e **As you rated this item highly please provide additional details to help us understand this challenge**

Please describe your experience of **Remote Working Arrangement** challenges on your research / research career. Please include whether the challenge was experienced before or after the COVID-19 pandemic was declared in March 2020 (or both).

________________________________________________________________

________________________________________________________________

________________________________________________________________

________________________________________________________________

________________________________________________________________

| Page Break |  |
| --- | --- |

*Display This Question:*

*If Please rate your experience of the following professional challenges you have encountered since t... [ Travel (e.g., regionally, nationally, or internationally for meetings, conferences etc) ] >= 7*

Q3f **As you rated this item highly please provide additional details to help us understand this challenge**

Please describe your experience of **Travel** challenges on your research / research career. Please include whether the challenge was experienced before or after the COVID-19 pandemic was declared in March 2020 (or both).

________________________________________________________________

________________________________________________________________

________________________________________________________________

________________________________________________________________

________________________________________________________________

| Page Break |  |
| --- | --- |

*Display This Question:*

*If Please rate your experience of the following professional challenges you have encountered since t... [ Supervision (e.g., type, frequency, etc) ] >= 7*

Q3g **As you rated this item highly please provide additional details to help us understand this challenge**

Please describe your experience of **Supervision** challenges on your research / research career. Please include whether the challenge was experienced before or after the COVID-19 pandemic was declared in March 2020 (or both).

________________________________________________________________

________________________________________________________________

________________________________________________________________

________________________________________________________________

________________________________________________________________

| Page Break |  |
| --- | --- |

*Display This Question:*

*If Please rate your experience of the following professional challenges you have encountered since t... [ Time management (e.g. time pressure to complete degree/projects on time or within funding window) ] >= 7*

Q3h **As you rated this item highly please provide additional details to help us understand this challenge**

Please describe your experience of **Time Management** challenges on your research / research career. Please include whether the challenge was experienced before or after the COVID-19 pandemic was declared in March 2020 (or both).

________________________________________________________________

________________________________________________________________

________________________________________________________________

________________________________________________________________

________________________________________________________________

| Page Break |  |
| --- | --- |

*Display This Question:*

*If Please rate your experience of the following professional challenges you have encountered since t... [ Research culture (e.g., distrust related to science, changing landscape) ] >= 7*

Q3i **As you rated this item highly please provide additional details to help us understand this challenge**

Please describe your experience of **Research Culture** challenges on your research / research career. Please include whether the challenge was experienced before or after the COVID-19 pandemic was declared in March 2020 (or both).

________________________________________________________________

________________________________________________________________

________________________________________________________________

________________________________________________________________

________________________________________________________________

| Page Break |  |
| --- | --- |

*Display This Question:*

*If Please rate your experience of the following professional challenges you have encountered since t... [ Other: please state ] >= 7*

Q3j **As you rated this item highly please provide additional details to help us understand this challenge**

Please describe your experience of **Other (${Q2/ChoiceTextEntryValue/10})**challenges on your research / research career. Please include whether the challenge was experienced before or after the COVID-19 pandemic was declared in March 2020 (or both).

________________________________________________________________

________________________________________________________________

________________________________________________________________

________________________________________________________________

________________________________________________________________

| Page Break |  |
| --- | --- |

Q4 Please rate your experience of the following **personal** challenges you have encountered since the COVID-19 pandemic was declared in March 2020, with 1 being no impact on your research / research career, and 10 being significant impact on your research / research career. Remember to consider only those personal challenges that may impact your research / professional capacity.

**This information is voluntary and confidential.** 

|  | No impact | Significant impact | Not Applicable |
| --- | --- | --- | --- |

|  | 1 | 2 | 3 | 4 | 5 | 6 | 7 | 8 | 9 | 10 |
| --- | --- | --- | --- | --- | --- | --- | --- | --- | --- | --- |

| Caregiving (e.g., having children or other caring responsibilities; lack of childcare) () | 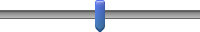 |
| --- | --- |
| Language (e.g., not being a native speaker to your study/work location) () | 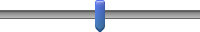 |
| Mental illness () | 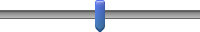 |
| Physical illness () | 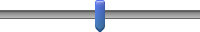 |
| Grief () | 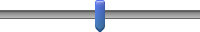 |
| Financial situation () | 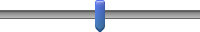 |
| Loneliness/homesickness () | 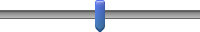 |
| Being infected with/exposed to COVID-19 () | 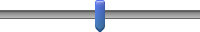 |
| Being in quarantine/ isolation () | 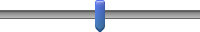 |
| Other: please state () | 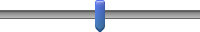 |

| Page Break |  |
| --- | --- |

*Display This Question:*

*If Please rate your experience of the following personal challenges you have encountered since the C... [ Caregiving (e.g., having children or other caring responsibilities; lack of childcare) ] >= 7*

Q5a **As you rated this item highly please provide additional details to help us understand this challenge**

If you are comfortable sharing, please describe your experience of **Caregiving** challenges on your research / research career. Please include whether the challenge was experienced before or after the COVID-19 pandemic was declared in March 2020 (or both).

________________________________________________________________

________________________________________________________________

________________________________________________________________

________________________________________________________________

________________________________________________________________

| Page Break |  |
| --- | --- |

*Display This Question:*

*If Please rate your experience of the following personal challenges you have encountered since the C... [ Language (e.g., not being a native speaker to your study/work location) ] >= 7*

Q5b **As you rated this item highly please provide additional details to help us understand this challenge**

If you are comfortable sharing, please describe your experience of **Language** challenges on your research / research career. Please include whether the challenge was experienced before or after the COVID-19 pandemic was declared in March 2020 (or both).

________________________________________________________________

________________________________________________________________

________________________________________________________________

________________________________________________________________

________________________________________________________________

| Page Break |  |
| --- | --- |

*Display This Question:*

*If Please rate your experience of the following personal challenges you have encountered since the C... [ Mental illness ] >= 7*

Q5c **As you rated this item highly please provide additional details to help us understand this challenge**

If you are comfortable sharing, please describe your experience of **Mental illness**challenges on your research / research career. Please include whether the challenge was experienced before or after the COVID-19 pandemic was declared in March 2020 (or both).

________________________________________________________________

________________________________________________________________

________________________________________________________________

________________________________________________________________

________________________________________________________________

| Page Break |  |
| --- | --- |

*Display This Question:*

*If Please rate your experience of the following personal challenges you have encountered since the C... [ Physical illness ] >= 7*

Q5d **As you rated this item highly please provide additional details to help us understand this challenge**

If you are comfortable sharing, please describe your experience of **Physical illness** challenges on your research / research career. Please include whether the challenge was experienced before or after the COVID-19 pandemic was declared in March 2020 (or both).

________________________________________________________________

________________________________________________________________

________________________________________________________________

________________________________________________________________

________________________________________________________________

| Page Break |  |
| --- | --- |

*Display This Question:*

*If Please rate your experience of the following personal challenges you have encountered since the C... [ Grief ] >= 7*

Q5e **As you rated this item highly please provide additional details to help us understand this challenge**

If you are comfortable sharing, please describe your experience of **Grief** challenges on your research / research career. Please include whether the challenge was experienced before or after the COVID-19 pandemic was declared in March 2020 (or both).

________________________________________________________________

________________________________________________________________

________________________________________________________________

________________________________________________________________

________________________________________________________________

| Page Break |  |
| --- | --- |

*Display This Question:*

*If Please rate your experience of the following personal challenges you have encountered since the C... [ Financial situation ] >= 7*

Q5f **As you rated this item highly please provide additional details to help us understand this challenge**

If you are comfortable sharing, please describe your experience of **Financial situation** challenges on your research / research career. Please include whether the challenge was experienced before or after the COVID-19 pandemic was declared in March 2020 (or both).

________________________________________________________________

________________________________________________________________

________________________________________________________________

________________________________________________________________

________________________________________________________________

| Page Break |  |
| --- | --- |

*Display This Question:*

*If Please rate your experience of the following personal challenges you have encountered since the C... [ Loneliness/homesickness ] >= 7*

Q5g **As you rated this item highly please provide additional details to help us understand this challenge**

If you are comfortable sharing, please describe your experience of**Loneliness/homesickness** challenges on your research / research career. Please include whether the challenge was experienced before or after the COVID-19 pandemic was declared in March 2020 (or both).

________________________________________________________________

________________________________________________________________

________________________________________________________________

________________________________________________________________

________________________________________________________________

| Page Break |  |
| --- | --- |

*Display This Question:*

*If Please rate your experience of the following personal challenges you have encountered since the C... [ Being infected with/exposed to COVID-19 ] >= 7*

Q5h **As you rated this item highly please provide additional details to help us understand this challenge**

If you are comfortable sharing, please describe your experience of **Being infected with/exposed to COVID-19** challenges on your research / research career. Please include whether the challenge was experienced before or after the COVID-19 pandemic was declared in March 2020 (or both).

________________________________________________________________

________________________________________________________________

________________________________________________________________

________________________________________________________________

________________________________________________________________

| Page Break |  |
| --- | --- |

*Display This Question:*

*If Please rate your experience of the following personal challenges you have encountered since the C... [ Being in quarantine/ isolation ] >= 7*

Q5i **As you rated this item highly please provide additional details to help us understand this challenge**

If you are comfortable sharing, please describe your experience of **Being in quarantine / isolation** challenges on your research / research career. Please include whether the challenge was experienced before or after the COVID-19 pandemic was declared in March 2020 (or both).

________________________________________________________________

________________________________________________________________

________________________________________________________________

________________________________________________________________

________________________________________________________________

| Page Break |  |
| --- | --- |

*Display This Question:*

*If Please rate your experience of the following personal challenges you have encountered since the C... [ Other: please state ] >= 7*

Q5j **As you rated this item highly please provide additional details to help us understand this challenge**

If you are comfortable sharing, please describe your experience of **Other** **(${Q4/ChoiceTextEntryValue/10}**) challenges on your research / research career. Please include whether the challenge was experienced before or after the COVID-19 pandemic was declared in March 2020 (or both).

________________________________________________________________

________________________________________________________________

________________________________________________________________

________________________________________________________________

________________________________________________________________

**End of Block: Challenges**

**Start of Block: Needs**

Information **Part 2. Needs**
The next questions focus on your needs both professionally and personally. When referring to personal challenges, think of these as challenges that impact your research / professional capacity.

Q6 Please rate your current needs for the following profes**sional dev**elopment areas, with 1 being no need for your research / research career, and 10 being significant need for your research / research career.

|  | No need | Significant need | Not Applicable |
| --- | --- | --- | --- |

|  | 1 | 2 | 3 | 4 | 5 | 6 | 7 | 8 | 9 | 10 |
| --- | --- | --- | --- | --- | --- | --- | --- | --- | --- | --- |

| Scientific writing (e.g., publications) () | 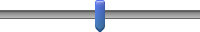 |
| --- | --- |
| Research methods / designs () | 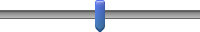 |
| Analysis (quantitative or qualitative) () | 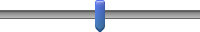 |
| Grant writing () | 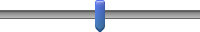 |
| Communicating research findings **outside of** academia (e.g. public, practitioners, policy makers) () | 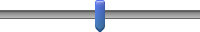 |
| Working with consumer researchers / end users () | 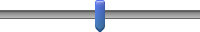 |
| Communicating research findings **within** academia () | 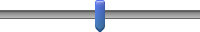 |
| Networking and collaboration opportunities () | 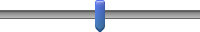 |
| Preparing job applications / resumes () | 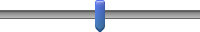 |
| Project management (including financial management of projects) () | 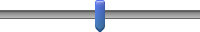 |
| Leadership () | 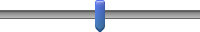 |
| Managing research staff () | 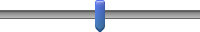 |
| Supervising research students () | 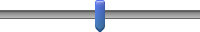 |
| Other: please state () | 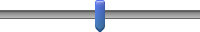 |

| Page Break |  |
| --- | --- |

*Display This Question:*

*If Please rate your current needs for the following professional development areas, with 1 being no... [ Scientific writing (e.g., publications) ] >= 7*

Q7a **As you rated this item highly please provide additional details to help us understand this need**

Please describe your needs for **Scientific writing (e.g., publications)**. Please include whether it was needed before or after the COVID-19 pandemic was declared in March 2020 (or both).

________________________________________________________________

________________________________________________________________

________________________________________________________________

________________________________________________________________

________________________________________________________________

| Page Break |  |
| --- | --- |

*Display This Question:*

*If Please rate your current needs for the following professional development areas, with 1 being no... [ Research methods / designs ] >= 7*

Q7b **As you rated this item highly please provide additional details to help us understand this need**

Please describe your needs for **Research methods / designs.** Please include whether it was needed before or after the COVID-19 pandemic was declared in March 2020 (or both).

________________________________________________________________

________________________________________________________________

________________________________________________________________

________________________________________________________________

________________________________________________________________

| Page Break |  |
| --- | --- |

*Display This Question:*

*If Please rate your current needs for the following professional development areas, with 1 being no... [ Analysis (quantitative or qualitative) ] >= 7*

Q7c **As you rated this item highly please provide additional details to help us understand this need**

Please describe your needs for **Analysis (quantitative or qualitative**). Please include whether it was needed before or after the COVID-19 pandemic was declared in March 2020 (or both).

________________________________________________________________

________________________________________________________________

________________________________________________________________

________________________________________________________________

________________________________________________________________

| Page Break |  |
| --- | --- |

*Display This Question:*

*If Please rate your current needs for the following professional development areas, with 1 being no... [ Grant writing ] >= 7*

Q7d **As you rated this item highly please provide additional details to help us understand this need**

Please describe your needs for **Grant writing**. Please include whether it was needed before or after the COVID-19 pandemic was declared in March 2020 (or both).

________________________________________________________________

________________________________________________________________

________________________________________________________________

________________________________________________________________

________________________________________________________________

| Page Break |  |
| --- | --- |

*Display This Question:*

*If Please rate your current needs for the following professional development areas, with 1 being no... [ Communicating research findings <strong><u>outside of</u> </strong>academia (e.g. public, practitioners, policy makers) ] >= 7*

Q7e **As you rated this item highly please provide additional details to help us understand this need**

Please describe your needs for **Communicating research findings outside of academia (e.g. public, practitioners, policy makers)**. Please include whether it was needed before or after the COVID-19 pandemic was declared in March 2020 (or both).

________________________________________________________________

________________________________________________________________

________________________________________________________________

________________________________________________________________

________________________________________________________________

| Page Break |  |
| --- | --- |

*Display This Question:*

*If Please rate your current needs for the following professional development areas, with 1 being no... [ Working with consumer researchers / end users ] >= 7*

Q7f **As you rated this item highly please provide additional details to help us understand this need**

Please describe your needs for **Working with consumer researchers / end users**. Please include whether it was needed before or after the COVID-19 pandemic was declared in March 2020 (or both).

________________________________________________________________

________________________________________________________________

________________________________________________________________

________________________________________________________________

________________________________________________________________

| Page Break |  |
| --- | --- |

*Display This Question:*

*If Please rate your current needs for the following professional development areas, with 1 being no... [ Communicating research findings <u><strong>within</strong></u> academia ] >= 7*

Q7g **As you rated this item highly please provide additional details to help us understand this need**

Please describe your needs for **Communicating research findings within academia**. Please include whether it was needed before or after the COVID-19 pandemic was declared in March 2020 (or both).

________________________________________________________________

________________________________________________________________

________________________________________________________________

________________________________________________________________

________________________________________________________________

| Page Break |  |
| --- | --- |

*Display This Question:*

*If Please rate your current needs for the following professional development areas, with 1 being no... [ Networking and collaboration opportunities ] >= 7*

Q7h **As you rated this item highly please provide additional details to help us understand this need**

Please describe your needs for **Networking and collaboration opportunities**. Please include whether it was needed before or after the COVID-19 pandemic was declared in March 2020 (or both).

________________________________________________________________

________________________________________________________________

________________________________________________________________

________________________________________________________________

________________________________________________________________

| Page Break |  |
| --- | --- |

*Display This Question:*

*If Please rate your current needs for the following professional development areas, with 1 being no... [ Preparing job applications / resumes ] >= 7*

Q7i **As you rated this item highly please provide additional details to help us understand this need**

Please describe your needs for **Preparing job applications / resumes**. Please include whether it was needed before or after the COVID-19 pandemic was declared in March 2020 (or both).

________________________________________________________________

________________________________________________________________

________________________________________________________________

________________________________________________________________

________________________________________________________________

| Page Break |  |
| --- | --- |

*Display This Question:*

*If Please rate your current needs for the following professional development areas, with 1 being no... [ Project management (including financial management of projects) ] >= 7*

Q7j **As you rated this item highly please provide additional details to help us understand this need**

Please describe your needs for **Project management (including financial management of projects)**. Please include whether it was needed before or after the COVID-19 pandemic was declared in March 2020 (or both).

________________________________________________________________

________________________________________________________________

________________________________________________________________

________________________________________________________________

________________________________________________________________

| Page Break |  |
| --- | --- |

*Display This Question:*

*If Please rate your current needs for the following professional development areas, with 1 being no... [ Leadership ] >= 7*

Q7k **As you rated this item highly please provide additional details to help us understand this need**

Please describe your needs for **Leadership**. Please include whether it was needed before or after the COVID-19 pandemic was declared in March 2020 (or both).

________________________________________________________________

________________________________________________________________

________________________________________________________________

________________________________________________________________

________________________________________________________________

| Page Break |  |
| --- | --- |

*Display This Question:*

*If Please rate your current needs for the following professional development areas, with 1 being no... [ Managing research staff ] >= 7*

Q7l **As you rated this item highly please provide additional details to help us understand this need**

Please describe your needs for **Managing research staff**. Please include whether it was needed before or after the COVID-19 pandemic was declared in March 2020 (or both).

________________________________________________________________

________________________________________________________________

________________________________________________________________

________________________________________________________________

________________________________________________________________

| Page Break |  |
| --- | --- |

*Display This Question:*

*If Please rate your current needs for the following professional development areas, with 1 being no... [ Supervising research students ] >= 7*

Q7m **As you rated this item highly please provide additional details to help us understand this need**

Please describe your needs for **Supervising research students**. Please include whether it was needed before or after the COVID-19 pandemic was declared in March 2020 (or both).

________________________________________________________________

________________________________________________________________

________________________________________________________________

________________________________________________________________

________________________________________________________________

| Page Break |  |
| --- | --- |

*Display This Question:*

*If Please rate your current needs for the following professional development areas, with 1 being no... [ Other: please state ] >= 7*

Q7n **As you rated this item highly please provide additional details to help us understand this need**

Please describe your needs for **Other (${Q6/ChoiceTextEntryValue/14})**. Please include whether it was needed before or after the COVID-19 pandemic was declared in March 2020 (or both).

________________________________________________________________

________________________________________________________________

________________________________________________________________

________________________________________________________________

________________________________________________________________

| Page Break |  |
| --- | --- |

Halfway message **Thank you for your responses so far.

You’re halfway through the survey!**

| Page Break |  |
| --- | --- |

Q8 Please rate your current needs for the following **personal development areas**, with 1 being no need for your research / research career, and 10 being needed for your research / research career.

*Remember to consider only those personal challenges relevant to your research / professional capacity.*

**This information is voluntary and confidential.**

|  | No need | Significant need | Not Applicable |
| --- | --- | --- | --- |

|  | 1 | 2 | 3 | 4 | 5 | 6 | 7 | 8 | 9 | 10 |
| --- | --- | --- | --- | --- | --- | --- | --- | --- | --- | --- |

| Stress and resilience () | 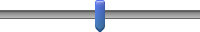 |
| --- | --- |
| Physical and emotional health management () | 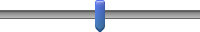 |
| Work-life balance () | 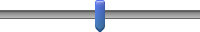 |
| Career planning () | 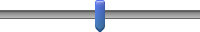 |
| Personal relationships with significant others () | 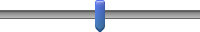 |
| Sleep and routine management () | 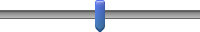 |
| Financial planning () | 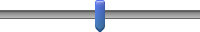 |
| Language () | 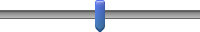 |
| Other: please state () | 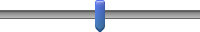 |

| Page Break |  |
| --- | --- |

*Display This Question:*

*If Please rate your current needs for the following personal development areas, with 1 being no need... [ Stress and resilience ] >= 7*

Q9a **As you rated this item highly please provide additional details to help us understand this need**

Please describe your current needs for **Stress and resilience**. Please include whether it was needed before or after the COVID-19 pandemic was declared in March 2020 (or both).

________________________________________________________________

________________________________________________________________

________________________________________________________________

________________________________________________________________

________________________________________________________________

| Page Break |  |
| --- | --- |

*Display This Question:*

*If Please rate your current needs for the following personal development areas, with 1 being no need... [ Physical and emotional health management ] >= 7*

Q9b **As you rated this item highly please provide additional details to help us understand this need**

Please describe your current needs for **Physical and emotional health management**. Please include whether it was needed before or after the COVID-19 pandemic was declared in March 2020 (or both).

________________________________________________________________

________________________________________________________________

________________________________________________________________

________________________________________________________________

________________________________________________________________

| Page Break |  |
| --- | --- |

*Display This Question:*

*If Please rate your current needs for the following personal development areas, with 1 being no need... [ Work-life balance ] >= 7*

Q9c **As you rated this item highly please provide additional details to help us understand this need**

Please describe your current needs for **Work-life balance**. Please include whether it was needed before or after the COVID-19 pandemic was declared in March 2020 (or both).

________________________________________________________________

________________________________________________________________

________________________________________________________________

________________________________________________________________

________________________________________________________________

| Page Break |  |
| --- | --- |

*Display This Question:*

*If Please rate your current needs for the following personal development areas, with 1 being no need... [ Career planning ] >= 7*

Q9d **As you rated this item highly please provide additional details to help us understand this need**

Please describe your current needs for **Career planning**. Please include whether it was needed before or after the COVID-19 pandemic was declared in March 2020 (or both).

________________________________________________________________

________________________________________________________________

________________________________________________________________

________________________________________________________________

________________________________________________________________

| Page Break |  |
| --- | --- |

*Display This Question:*

*If Please rate your current needs for the following personal development areas, with 1 being no need... [ Personal relationships with significant others ] >= 7*

Q9e **As you rated this item highly please provide additional details to help us understand this need**

Please describe your current needs for **Personal relationships with significant others**. Please include whether it was needed before or after the COVID-19 pandemic was declared in March 2020 (or both).

________________________________________________________________

________________________________________________________________

________________________________________________________________

________________________________________________________________

________________________________________________________________

| Page Break |  |
| --- | --- |

*Display This Question:*

*If Please rate your current needs for the following personal development areas, with 1 being no need... [ Sleep and routine management ] >= 7*

Q9f **As you rated this item highly please provide additional details to help us understand this need**

Please describe your current needs for **Sleep and routine management**. Please include whether it was needed before or after the COVID-19 pandemic was declared in March 2020 (or both).

________________________________________________________________

________________________________________________________________

________________________________________________________________

________________________________________________________________

________________________________________________________________

| Page Break |  |
| --- | --- |

*Display This Question:*

*If Please rate your current needs for the following personal development areas, with 1 being no need... [ Financial planning ] >= 7*

Q9g **As you rated this item highly please provide additional details to help us understand this need**

Please describe your current needs for **Financial planning**. Please include whether it was needed before or after the COVID-19 pandemic was declared in March 2020 (or both).

________________________________________________________________

________________________________________________________________

________________________________________________________________

________________________________________________________________

________________________________________________________________

| Page Break |  |
| --- | --- |

*Display This Question:*

*If Please rate your current needs for the following personal development areas, with 1 being no need... [ Language ] >= 7*

Q9h **As you rated this item highly please provide additional details to help us understand this need**

Please describe your current needs for **Language**. Please include whether it was needed before or after the COVID-19 pandemic was declared in March 2020 (or both).

________________________________________________________________

________________________________________________________________

________________________________________________________________

________________________________________________________________

________________________________________________________________

| Page Break |  |
| --- | --- |

*Display This Question:*

*If Please rate your current needs for the following personal development areas, with 1 being no need... [ Other: please state ] >= 7*

Q9i **As you rated this item highly please provide additional details to help us understand this need**

Please describe your current needs for **Other ${q://QID65/ChoiceTextEntryValue/9**}. Please include whether it was needed before or after the COVID-19 pandemic was declared in March 2020 (or both).

________________________________________________________________

________________________________________________________________

________________________________________________________________

________________________________________________________________

________________________________________________________________

**End of Block: Needs**

**Start of Block: Opportunities**

Information **Part 3. Opportunities**

You are more than halfway through completing the survey!

The next questions focus on the opportunities for professional and personal development you currently have access to and would like to have in the future.

| Page Break |  |
| --- | --- |

Q10 Please outline professional development opportunities that you currently have access to and how they benefit your **professional development** (e.g., writing retreats, courses).

________________________________________________________________

________________________________________________________________

________________________________________________________________

________________________________________________________________

________________________________________________________________

Q11 Please outline professional development opportunities that you currently have access to and how they benefit your **personal development** (e.g., mentoring, peer support).

________________________________________________________________

________________________________________________________________

________________________________________________________________

________________________________________________________________

________________________________________________________________

| Page Break |  |
| --- | --- |

Q12 Please outline the professional development opportunities you would like to **have access to in the future?**
 *Please include any details regarding topic, structure, delivery format.*

________________________________________________________________

________________________________________________________________

________________________________________________________________

________________________________________________________________

________________________________________________________________

Q13 Please provide detail of the professional development opportunities you currently have access to that you would **highly recommend** to other emerging researchers.
 *Please include the name of the opportunity and provider or links to further information.*

________________________________________________________________

________________________________________________________________

________________________________________________________________

________________________________________________________________

________________________________________________________________

| Page Break |  |
| --- | --- |

Information This final question is very broad and seeking your imaginative perspective on what the future may look like for emerging researchers in the behavioral nutrition and physical activity research field.

Q14 What do you see as the **opportunities for your research / research career** into the future (i.e., new normal/post-pandemic norm)?
 *Please interpret the term ‘opportunities’ very broadly, there is no limit to the scope with this question. (e.g. broadening the research scope and knowledge, increasing communication effectiveness through online meetings, advances in technologies, experimenting with new research ideas and methodologies to cope with the new normal etc.)*

________________________________________________________________

________________________________________________________________

________________________________________________________________

________________________________________________________________

________________________________________________________________

**End of Block: Opportunities**

**Start of Block: Demographics**

information PART 4. ABOUT YOU 
Great work - you've made it to the final section of the survey!
 
The final questions in this section focus on your demographics and career stage.

Q15 What is your age?

- Please enter your age (in years): (1) __________________________________________________
- Prefer not to answer (2)

Q16 Which gender do you identify as?

- Woman (1)
- Man (2)
- Non-binary (3)
- Prefer to self-describe: (4) __________________________________________________
- Prefer not to answer (5)

Q17 There is substantial variation in how people think about race and ethnicity around the world. Do any of these response options describe your racial or ethnic identity? If so, select all that apply.
If not, please provide your racial or ethnic identity in “Other.”
If you do not have a racial or ethnic identity, select “Non-identifying.” 

- Asian (3)
- Black (4)
- Hispanic or Latino (5)
- Middle Eastern or North African (6)
- Native or Other Indigenous Peoples (7)
- Pacific Islander (8)
- White or Caucasian (9)
- Other - please specify (10) __________________________________________________
- Non-identifying (11)
- Prefer not to answer (2)

| Page Break |  |
| --- | --- |

| 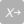 |
| --- |

Q18 In which country do you currently reside?

▼ Afghanistan (1) ... Zimbabwe (1357)

| 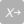 |
| --- |

Q19 How comfortable is your current financial situation?

 *Please rate on the scale below, from 'very uncomfortable' to 'very comfortable'*

|  | Very Uncomfortable (1) | Somewhat Uncomfortable (2) | Moderately Comfortable (3) | Somewhat Comfortable (4) | Very Comfortable (5) | Prefer not to answer (0) |
| --- | --- | --- | --- | --- | --- | --- |
| Rate your the comfort of your financial situation (1) |  |  |  |  |  |  |

Q20 What best describes your primary employment situation? Select as many that apply:

- Studying (with no scholarship) (1)
- Studying (with partial scholarship) (7)
- Studying (with full scholarship) (2)
- Employed (casually) (3)
- Employed (on fixed-term / short-term contract) (8)
- Employed (ongoing / permanent position) (9)
- Looking for Work (4)
- On Career Break/Disruption (5)
- Other (please specify): (6) __________________________________________________

| Page Break |  |
| --- | --- |

information **The following questions relate to your career stage.**

Q21 Which best describes your current career stage?

- Honour's Student/Undergraduate Student (with research component) (1)
- Master's Student (with research component) (2)
- PhD Student (3)
- Early Career Researcher (e.g., postdoctoral researcher, research fellow, faculty) (4)
- Other/Between Stages (please describe): (5) __________________________________________________

| Page Break |  |
| --- | --- |

*Display This Question:*

*If Which best describes your current career stage? = PhD Student*

Q21a What year did you begin your PhD?

________________________________________________________________

*Display This Question:*

*If Which best describes your current career stage? = Early Career Researcher (e.g., postdoctoral researcher, research fellow, faculty)*

Q21b What is your current role?

________________________________________________________________

*Display This Question:*

*If Which best describes your current career stage? = Early Career Researcher (e.g., postdoctoral researcher, research fellow, faculty)*

Q21c How many full-time equivalent years post-PhD are you (excluding career disruptions, teaching only roles, part-time commitments outside of research, etc.)?

________________________________________________________________

| Page Break |  |
| --- | --- |

Q22 What is your research field? (Select as many that apply)

- Nutrition (1)
- Physical Activity (2)
- Sedentary Behavior (3)
- Sleep (4)

Q23 What is the area you would like to work in? (i.e., career planning, post-training)

- Mostly Research (1)
- Teaching and Research Combination (2)
- Mostly Teaching (3)
- Industry (4)
- Government (5)
- Non-government organization (8)
- Unsure at this stage (6)
- Other (please specify): (7) __________________________________________________

Q24 Are you a member of the International Society of Behavioral Nutrition and Physical Activity (ISBNPA)?

- Yes, I am presently a member (1)
- No, but I have previously been a member (2)
- No, I have never been a member (3)

Q104 How did you hear about this research project?

- Twitter (1)
- Facebook (2)
- ISBNPA email (3)
- A friend or colleague (4)
- Conference or Course Presentation (5)
- UK SBM (6)
- ISPAH (7)
- INTUE (8)
- ASPA (10)
- NASPEM / PWP (11)
- ACSM (12)
- CSEP (13)
- Other (9) __________________________________________________

**End of Block: Demographics**

**Start of Block: Thankyou**

Thank you for completing this study.

A summary of findings from this study will be available on the [ISBNPA NESI Webpage](https://isbnpa.org/membership/nesi-website/) in 2023.

 Please click next to submit your responses and be redirected to another survey link to provide your email address if you would like to enter the prize draw to win a 2023 International Society of Behavioral Nutrition and Physical Activity annual membership, or to be contacted about future similar studies you may be eligible to participate in.

**End of Block: Thankyou**
