# Supplementary material for: Contemporary challenges, needs and opportunities for emerging behavioral nutrition and physical activity researchers: a mixed-methods study
Source: Int J Behav Nutr Phys Act. 2025 Jul 6;22:94. doi: 10.1186/s12966-025-01748-1 (PMC12232601; doi:10.1186/s12966-025-01748-1)
Supplement: Supplementary file 3 — Supplementary Material 3. [file 12966_2025_1748_MOESM3_ESM.docx]

**Additional file 3**

**Table S1.** Summary of participant characteristics

| **Characteristics** | | **n** |
| --- | --- | --- |
| Age (yrs, Mean, SD*) (n=110) | | 33.0, 7.1* |
| Gender (n=111) | |  |
|  | Man | 21 |
|  | Woman | 86 |
|  | Non-binary | 1 |
|  | Prefer not to answer | 3 |
| Race/Ethnicity (n=110) | |  |
|  | White | 80 |
|  | Hispanic or Latino only | 4 |
|  | Asian | 16 |
|  | Black | 4 |
|  | Other | 4 |
|  | Prefer not to answer | 3 |
| Continent of current residence (n=104) | |  |
|  | North America | 33 |
|  | South America | 4 |
|  | Europe | 26 |
|  | Asia | 4 |
|  | Africa | 4 |
|  | Oceania | 33 |
| Financial comfort (n=109) | |  |
|  | Very uncomfortable | 9 |
|  | Somewhat uncomfortable | 22 |
|  | Moderately comfortable | 27 |
|  | Somewhat comfortable | 34 |
|  | Very comfortable | 17 |
| Career stage (n=110) | |  |
|  | Masters student | 2 |
|  | PhD student | 61 |
|  | Early Career Researcher | 47 |
| Primary employment situation (n=109) | |  |
|  | Studying (with or without scholarship) | 55 |
|  | Employed (casually) | 18 |
|  | Employed (fixed-term / short term contract) | 42 |
|  | Employed (ongoing / permanent) | 24 |
|  | Looking for work | 4 |
|  | Other (including career break / disruption) | 4 |
| Field of Expertise^a^ | |  |
|  | Nutrition | 43 |
|  | Physical activity | 78 |
|  | Sedentary behaviour | 46 |
|  | Sleep | 20 |

^a^ Frequencies do not equal 100% because the item was ‘Select all that apply’.
