# Supplementary material for: Contemporary challenges, needs and opportunities for emerging behavioral nutrition and physical activity researchers: a mixed-methods study
Source: Int J Behav Nutr Phys Act. 2025 Jul 6;22:94. doi: 10.1186/s12966-025-01748-1 (PMC12232601; doi:10.1186/s12966-025-01748-1)
Supplement: Supplementary file 4 — Supplementary Material 4. [file 12966_2025_1748_MOESM4_ESM.docx]

**Additional file 4**

**Table S2.** Multivariable regression analyses examining socio-demographic predictors of professional development needs (n=103) and personal development needs (n=101)

| **Domains** | | **Coefficient** | **Standard Error** | ***t*-value** | **95% CI** | ***p*-value** |
| --- | --- | --- | --- | --- | --- | --- |
| **Professional development needs^1^** | |  |  |  |  |  |
|  | Age | -0.01 | 0.03 | -0.37 | -0.06, 0.04 | 0.71 |
|  | Gender (ref.= Man) |  |  |  |  |  |
|  | Woman | 0.61 | 0.46 | 1.33 | -0.30, 1.52 | 0.19 |
|  | Non-Binary | -0.30 | 2.50 | -0.12 | -5.28, 4.67 | 0.90 |
|  | Prefer not to answer | 3.33 | 2.18 | 1.53 | -1.00, 7.66 | 0.13 |
|  | Race/Ethnicity (ref.=White/Caucasian Only) | |  |  |  |  |
|  | Prefer not to answer | 0.39 | 1.74 | 0.22 | -3.07, 3.86 | 0.82 |
|  | **Hispanic/Latino Only** | **-2.99** | **1.26** | **-2.37** | **-5.49, -0.48** | **0.02** |
|  | Asian | 1.05 | 0.56 | 1.87 | -0.07, 2.17 | 0.07 |
|  | Black | 1.75 | 0.94 | 1.85 | -0.13, 3.62 | 0.07 |
|  | Other | -0.81 | 1.09 | -0.74 | -2.98, 1.36 | 0.46 |
|  | Continent (ref.=North America) | |  |  |  |  |
|  | **South America** | **2.45** | **1.08** | **2.27** | **0.30, 4.60** | **0.03** |
|  | Europe | -0.08 | 0.48 | -0.16 | -1.03, 0.88 | 0.87 |
|  | Asia | 1.02 | 0.98 | 1.04 | -0.93, 2.96 | 0.30 |
|  | Africa | 0.82 | 1.17 | 0.70 | -1.51, 3.16 | 0.48 |
|  | Oceania | 0.22 | 0.44 | 0.49 | -0.66, 1.10 | 0.62 |
|  | **Financial Situation** | **-0.33** | **0.16** | **-2.06** | **-0.65, -0.01** | **0.04** |
|  | Career Stage | -0.11 | 0.37 | -0.30 | -0.83, 0.62 | 0.77 |
|  | **Personal development needs^2^** |  |  |  |  |  |
| **Domains** | | **Coefficient** | **Standard Error** | ***t*-value** | **95% CI** | ***p*-value** |
|  | Age | -0.03 | 0.03 | -1.04 | -0.09, 0.03 | 0.30 |
|  | Gender (ref.= Man) |  |  |  |  |  |
|  | Woman | 0.53 | 0.52 | 1.02 | -0.50, 1.57 | 0.31 |
|  | Non-Binary | 0.78 | 2.84 | 0.27 | -4.87, 6.42 | 0.78 |
|  | Prefer not to answer | 1.68 | 2.49 | 0.68 | -3.26, 6.62 | 0.50 |
|  | Race/Ethnicity (ref.=White/Caucasian Only) | |  |  |  |  |
|  | Prefer not to answer | 2.32 | 1.98 | 1.17 | -1.61, 6.25 | 0.24 |
|  | Hispanic/Latino Only | 0.17 | 1.43 | 0.12 | -2.67, 3.01 | 0.91 |
|  | Asian | 0.24 | 0.66 | 0.37 | -1.07, 1.55 | 0.71 |
|  | Black | 0.77 | 1.22 | 0.63 | -1.66, 3.20 | 0.53 |
|  | Other | -1.67 | 1.24 | -1.35 | -4.14, 0.79 | 0.18 |
|  | Continent (ref.=North America) | |  |  |  |  |
|  | South America | 1.86 | 1.23 | 1.52 | -0.58, 4.30 | 0.13 |
|  | Europe | 0.27 | 0.54 | 0.50 | -0.81, 1.36 | 0.62 |
|  | Asia | -0.80 | 1.11 | -0.71 | -3.01, 1.42 | 0.48 |
|  | Africa | 0.54 | 1.38 | 0.39 | -2.21, 3.29 | 0.70 |
|  | Oceania | 0.04 | 0.51 | 0.08 | -0.97, 1.05 | 0.94 |
|  | **Financial Situation** | **-0.66** | **0.18** | **-3.60** | **-1.03, -0.30** | **<0.01** |
|  | Career Stage | -0.23 | 0.41 | -0.55 | -1.05, 0.59 | 0.58 |

^1^ Model fit statistics (F(86) = 1.99, p < .05, Adj. R2 = .13). Note. Significant predictors (p<0.05) are **bolded**.

^2^ Model fit statistics (F(84) = 1.72, p < .05, Adj. R2 = .10). Note. Significant predictors (p<0.05) are **bolded**.

**Table S3.** Multivariable regression analyses exploring socio-demographic predictors of professional challenges and personal challenges

| **Domains** | | **Coefficient** | **Standard Error** | ***t*-value** | **95% CI** | ***p*-value** | ***F*-statistic** | **Adjusted R^2^** |
| --- | --- | --- | --- | --- | --- | --- | --- | --- |
| **Professional Challenges** (n=103) | | - | - | - | - | 0.74 | 0.75 | -0.04 |
|  | Age | 0.03 | 0.03 | 1.30 | -0.02, 0.09 | 0.20 | - | - |
|  | Gender (ref.= Man) |  |  |  |  |  |  |  |
|  | Woman | 0.18 | 0.47 | 0.38 | -0.75, 1.11 | 0.70 | - | - |
|  | Non-Binary | 1.01 | 2.23 | 0.45 | -3.42, 5.43 | 0.65 | - | - |
|  | Prefer not to answer | -0.91 | 2.56 | -0.35 | -5.99, 4.18 | 0.72 | - | - |
|  | Race/Ethnicity (ref.=White/Caucasian Only) | |  |  |  |  |  |  |
|  | Prefer not to answer | 2.99 | 1.78 | 1.68 | -0.55, 6.54 | 0.10 | - | - |
|  | Hispanic/Latino Only | 1.66 | 1.29 | 1.29 | -0.91, 4.22 | 0.20 | - | - |
|  | Asian | 0.05 | 0.57 | 0.09 | -1.09, 1.19 | 0.93 | - | - |
|  | Black | 0.14 | 0.96 | 0.15 | -1.77, 2.06 | 0.88 | - | - |
|  | Other | 0.42 | 1.12 | 0.38 | -1.80, 2.64 | 0.71 | - | - |
|  | Continent (ref.=North America) | |  |  |  |  |  |  |
|  | South America | -0.36 | 1.11 | -0.33 | -2.56, 1.83 | 0.74 | - | - |
|  | Europe | -0.17 | 0.49 | -0.35 | -1.15, 0.80 | 0.73 | - | - |
|  | Asia | -0.24 | 1.00 | -0.24 | -2.23, 1.75 | 0.81 | - | - |
|  | Africa | -0.10 | 1.20 | -0.09 | -2.50, 2.28 | 0.93 | - | - |
|  | Oceania | -0.33 | 0.45 | -0.73 | -1.23, 0.57 | 0.47 | - | - |
|  | Financial Situation | -0.16 | 0.16 | -0.97 | -0.49, 0.17 | 0.33 | - | - |
|  | Career Stage | -0.03 | 0.37 | -0.08 | -0.77, 0.71 | 0.94 | - | - |
| **Personal Challenges** (n=102) | | - | - | - | - | 1.99 | 1.27 | 0.04 |
|  | Age | 0.01 | 0.03 | 0.29 | -0.05, 0.07 | 0.78 | - | - |
|  | Gender (ref.= Man) |  |  |  |  |  |  |  |
|  | Woman | 0.61 | 0.61 | 1.20 | -0.41, 1.63 | 0.23 | - | - |
|  | Non-Binary | 4.30 | 2.44 | 1.76 | -8.02, 3.15 | 0.39 | - | - |
|  | Prefer not to answer | -2.44 | 2.80 | -0.87 | -0.56, 9.15 | 0.08 | - | - |
|  | Race/Ethnicity (ref.=White/Caucasian Only) | |  |  |  |  |  |  |
|  | Prefer not to answer | 2.17 | 1.96 | 1.11 | -1.71, 6.06 | 0.27 | - | - |
|  | **Hispanic/Latino Only** | **2.93** | **1.41** | **2.07** | **0.12, 5.74** | **0.04** | - | - |
|  | Asian | 0.57 | 0.65 | 0.88 | -0.72, 1.86 | 0.38 | - | - |
|  | Black | 0.97 | 1.06 | 0.91 | -1.14, 3.07 | 0.36 | - | - |
|  | Other | -0.48 | 1.22 | -0.39 | -2.91, 1.95 | 0.70 | - | - |
|  | Continent (ref.=North America) | |  |  |  |  |  |  |
|  | South America | -0.55 | 1.21 | -0.45 | -2.96, 1.86 | 0.65 | - | - |
|  | Europe | -0.10 | 0.54 | -0.17 | -1.15, 0.98 | 0.87 | - | - |
|  | Asia | 0.92 | 1.10 | 0.84 | -1.27, 3.11 | 0.40 | - | - |
|  | Africa | -0.15 | 1.32 | -0.12 | -2.78, 2.47 | 0.90 | - | - |
|  | Oceania | -0.06 | 0.50 | -0.13 | -1.06, 0.93 | 0.90 | - | - |
|  | Financial Situation | -0.26 | 0.18 | -1.45 | -0.62, 0.10 | 0.15 | - | - |
|  | Career Stage | -0.58 | 0.41 | -1.43 | -1.40, 0.23 | 0.16 | - | - |

Note. Significant predictors (p<0.05) are bolded.
